# A Solitary Fibrous Tumor of the Pleura Revealed by Hiccups

**DOI:** 10.1155/2011/574319

**Published:** 2011-09-25

**Authors:** A. Chafik, M. Alaoui, A. Benjelloune, Y. Qamouss

**Affiliations:** ^1^Department of Thoracic Surgery, Avicenne Military Hospital, Marrakech 40000, Morocco; ^2^Cadi Ayyad University, Marrakech, Morocco; ^3^Department of Vascular Surgery, Avicenne Military Hospital, Marrakech 40000, Morocco; ^4^Department of Pulmonology, Avicenne Military Hospital, Marrakech 40000, Morocco; ^5^Department of Anaesthesiology, Avicenne Military Hospital, Marrakech 40000, Morocco

## Abstract

Solitary fibrous tumors of the pleura are rare and benign primary localized tumors; they possess a malignant potential and thus should be excised. We report a case of a 43-year-old woman, who had suffered for 5 years from right basithoracic pain associated with progressive dyspnea and persistent hiccups during the last 6 months. We have not found any similar case in the literature. Further testing after excision by thoracotomy revealed a solitary fibrous pleural tumor. A brief discussion of the clinical presentation and incidence of these tumors is included.

## 1. Introduction

Solitary fibrous tumors of the pleura are rare and benign primary localized tumors; only 800 cases have been reported in the literature so far [1]. These asymptomatic tumors occur at all ages and are usually discovered incidentally during a systematic chest X-ray. Sarcomatous degeneration is rare. Surgery can provide definitive diagnosis and excision of the mass.

 We report the case of a solitary fibrous tumor of the pleura revealed by hiccups and for which we have not found any similar case in the literature.

## 2. Observation

The patient was a 43-year-old woman, with no particular medical history, who had suffered for 5 years from right basithoracic pain associated with progressive dyspnea and persistent hiccups during the last 6 months. Pleuro-pulmonary examination revealed condensation signs on the right side. Blood count cells, serum electrolytes, and hemostasis were normal. The lung function showed a Forced Expiratory Volume in 1 second (FEV1) at 52% of the normal value and a Vital Capacity (VC) at 56%.

**Figure 1:**
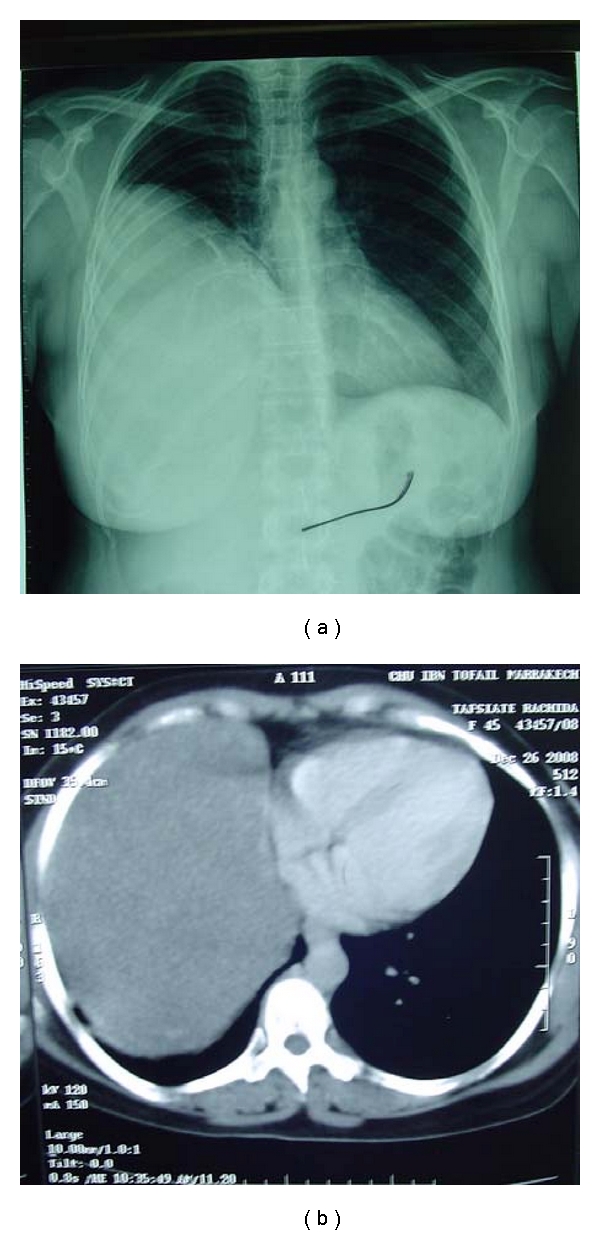
(a) Initial chest radiography showed right basithoracic mass with clear limits and obtuse connection angles. (b) Initial computed tomogram with contrast revealed one mild homogeneous, well-defined mass that compressed the mediastinum to left side.

The chest radiograph revealed a homogeneous, dense, right basithoracic mass with clear limits and obtuse connection angles (Figure 1(a)). The chest CT scan both revealed one huge tumor that occupied the right basithoracic cavity, approximately 25 cm in diameter, with chest wall distention and shifting of the mediastinum to the left side (Figure 1(b)). 

The CT-guided biopsy of the mass revealed a nonatypical spindle cells tumor. The patient underwent surgery by right posterolateral thoracotomy through the 5th intercostal space.

**Figure 2:**
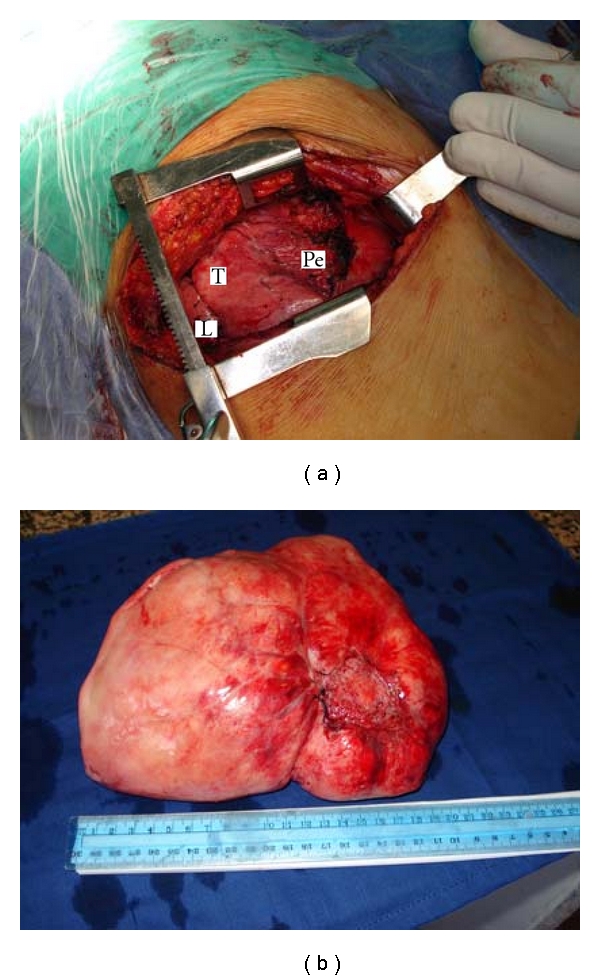
(a) Intraoperative photograph showing the characteristic pedicle (Pe) of a large solitary fibrous tumour of the pleura. L: lung; T: tumour. (b) The giant solitary fibrous tumor was well circumscribed, white, and firm, with a whorled appearance and focal necrosis on cut surface, and was approximately 25 cm in diameter.

Operatory exploration showed a well-limited, pedicled huge tissue mass. The lesion had developed at the expense of the middle lobe visceral pleura, adherent in places to the chest wall and diaphragm, displacing the lung and the mediastinum, with atelectasis of the right lower lobe and the middle lobe (Figure 2(a)). The complete releasing of the tumor from the diaphragm and the rest of the chest wall required the further opening of the 8th intercostal space by the same thoracotomy. The tumor's resection was complete with 1 cm margin (Figure 2(b)). It weighed 1.850 kg. Histological findings upheld the benign and fibrous nature of the tumor as well as its origin from the visceral pleura. Hiccups disappeared completely early postoperatively. The patient was discharged 8 days after surgery. Six months later, we noticed a significant decrease of the dyspnea, as well as an improvement of the lung function, with an FEV 1 at 76% and a VC at 71% of the normal values. The current hindsight without recurrence is 18 months.

## 3. Discussion

The solitary fibrous tumors of the pleura are tumors of mesenchymal origin. These tumors were described for the first time by Klemperer and Rabin in 1931 [2]. They represent less than 5% of all pleural tumors [3]. These lesions are derived from the visceral pleura in two-thirds of cases and from the parietal pleura in one-third of cases [3]. They are rarely intrapulmonary or pericardial and exceptionally extrathoracic (thyroid, orbit, nasopharynx, nose, and meninges) [4]. The solitary fibrous tumors of the pleura occur at all ages (5–87 years) with a peak in the 6th and 7th decades; they affect equally men and women [4]. The usual presentation is an incidental discovery on chest radiograph (54% of cases) [3]. The most common symptoms are dyspnea, cough, and chest pain. Paraneoplastic signs such as hypertrophic osteoarthropathy syndrome (Pierre-Marie-Lambert) related to the secretion of hyaluronic acid (20% of cases), and refractory hypoglycemia (Doege-Potter syndrome) due to insulin hypersecretion (5% of cases) may be associated [5].

Cases revealed by edema of the lower limbs due to the compression of the right atrium and vena cava [6] or by syncope caused by coughing and phrenic nerve stimulation [5] have also been reported. To our knowledge no cases have been revealed by medication resistant hiccups.

The diagnosis can be suspected on a chest radiograph. The size can vary from one centimeter to the entire hemi-field lung. The chest CT scan allows a quite accurate study of the tumor, as it indicates its boundaries and its relationship to adjacent organs. It also allows the tumor's resectability evaluation, showing its displacement and noninvasion of the surrounding structures. These tumors are lobulated and are of heterogeneous density. The thoracic MRI can be of great help in case of imprecise data on the CT scan. Transparietal puncture is often inconclusive, with a risk of dissemination on the route of the needle. It is rather indicated for large, unresectable, and potentially aggressive tumors.

These tumors were initially classified as mesothelial localized tumors. Fibroblastic characteristics were studied by immunohistochemistry and ultrastructure. The expression by these tumors of vimentin and CD-34, which are markers of mesenchymal cells, and negativity to cytoplasmic keratin indicates that these fibromas are of mesenchymal origin [3]. 

These tumors also express Bcl-2 and b-catenin. The criteria for malignancy remain controversial. For England et al. [7], malignancy is suspected in case of high mitotic index (more than 4 mitoses per 10 fields), presence of hemorrhage, increased cellularity, pleomorphism, and necrosis.

For other authors [8], infiltration of adjacent structures is necessary to assert malignancy. For Carretta et al. [9], the histological criteria of malignancy are sessile implantation on the parietal pleura and negative immunoreactivity for progesterone receptors. Presence of abnormalities on the long arm of chromosome 9 is suspected to be involved in the pathogenesis of these tumors [10].

The main treatment is surgery. England et al. [7] have reviewed 223 cases of solitary fibrous tumors of the pleura and demonstrated that complete resection is the main factor for long-term survival. The benign lesions have had a recurrence rate of 8% compared to malignant lesions, with a 63% relapse rate after resection [1]. In case of recurrence, surgical resection is indicated in the absence of metastasis.

Radio-chemotherapy may be indicated in case of malignant tumors with metastases. Thalidomide has anti-angiogenic activity by inhibition of the vascular endothelial growth factor and the fibroblast growth factor. It has been prescribed as adjuvant therapy for malignant solitary fibrous tumors with good results [3].

## 4. Conclusion

The solitary fibrous tumors of the pleura are rare lesions, usually of fortuitous discovery. Hiccups can be a revealing symptom. Their evolution is generally benign, and surgery is the main treatment.
